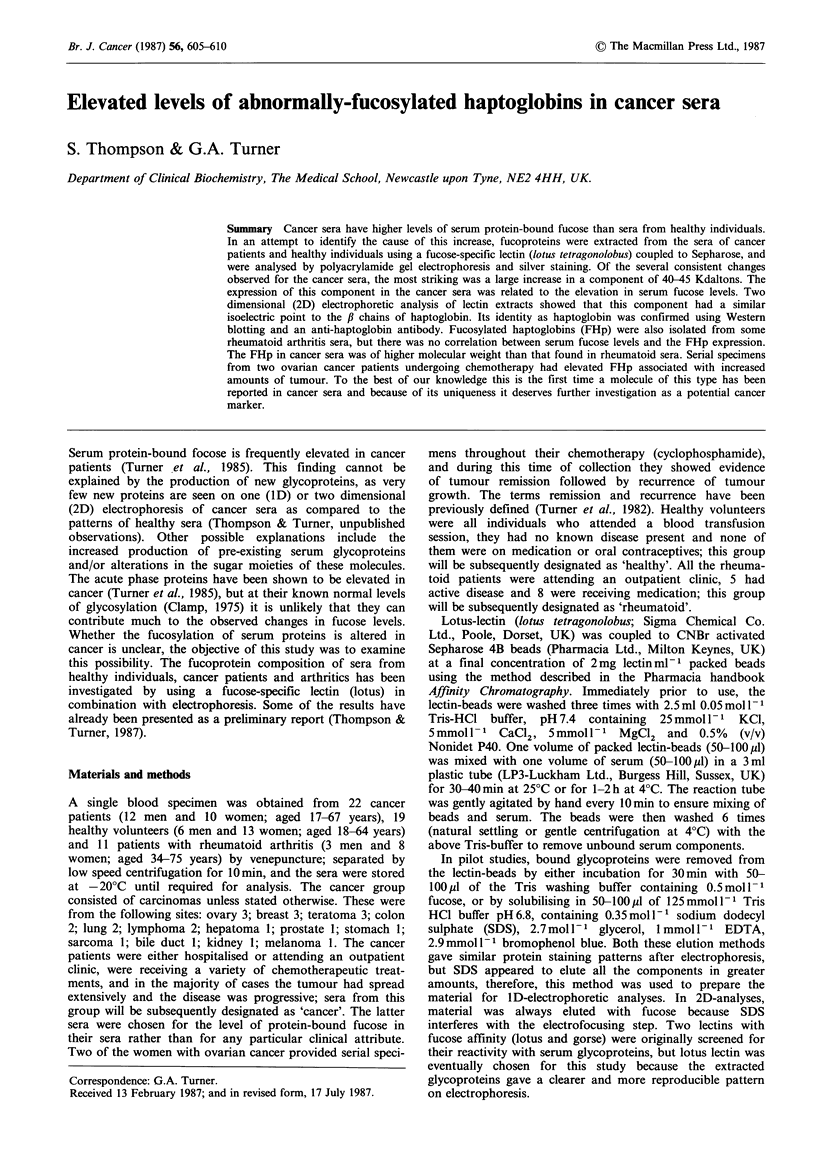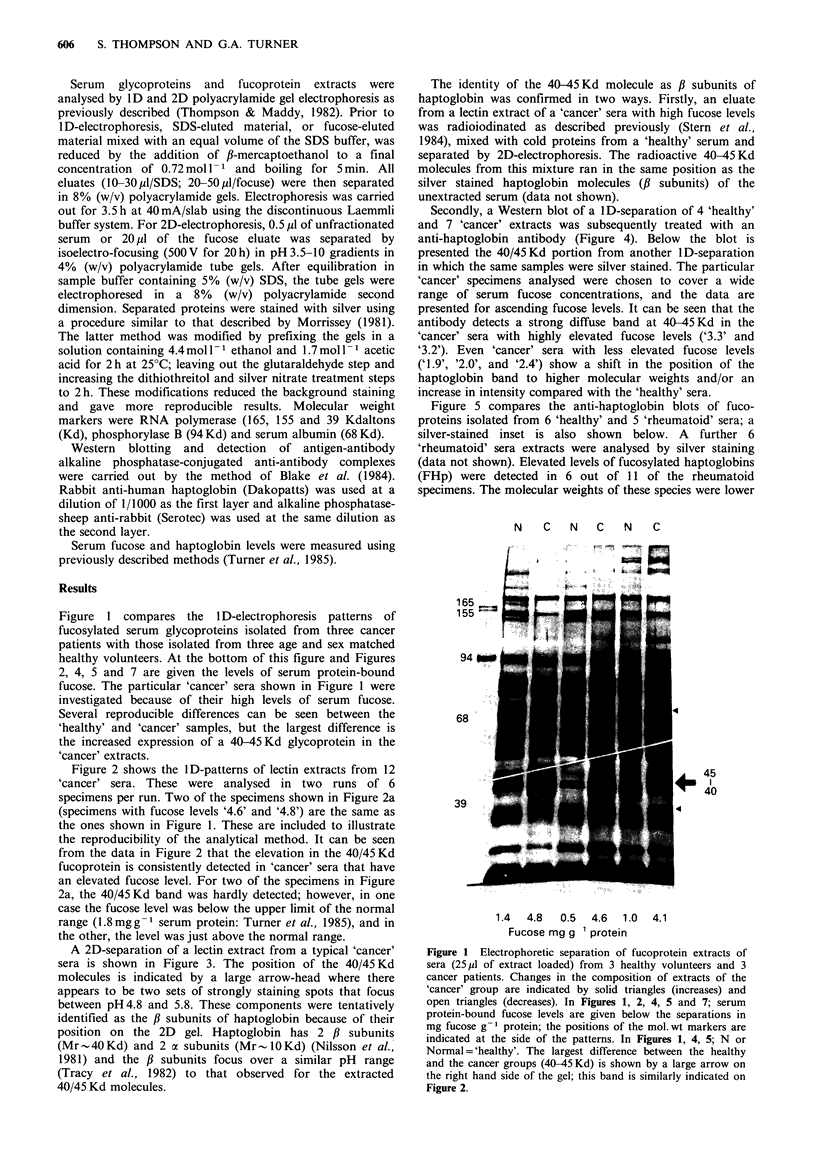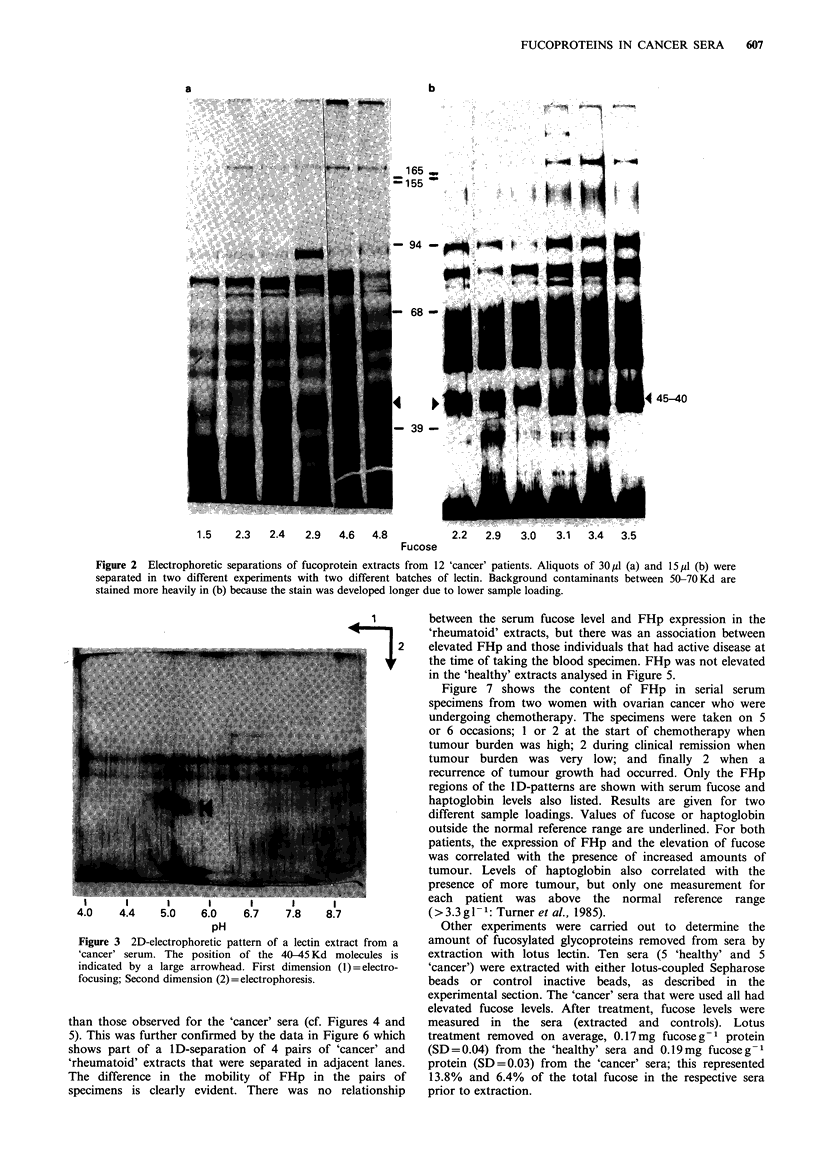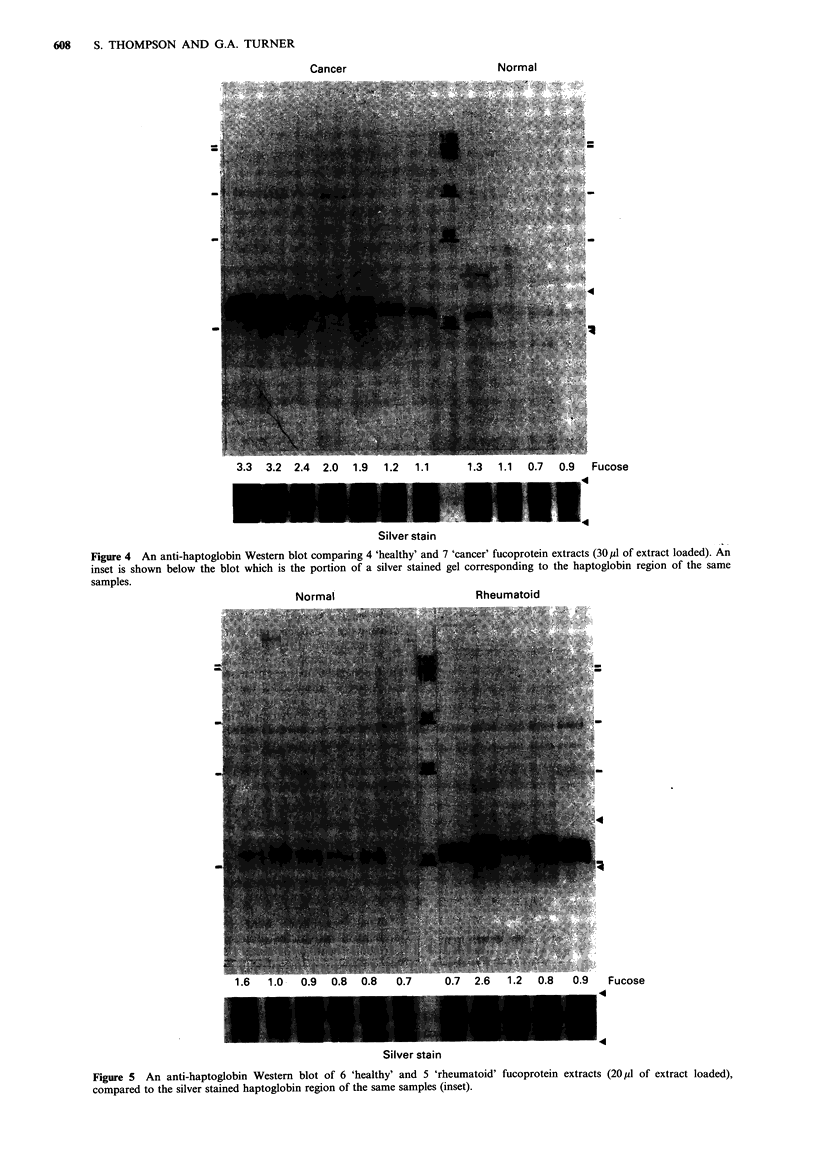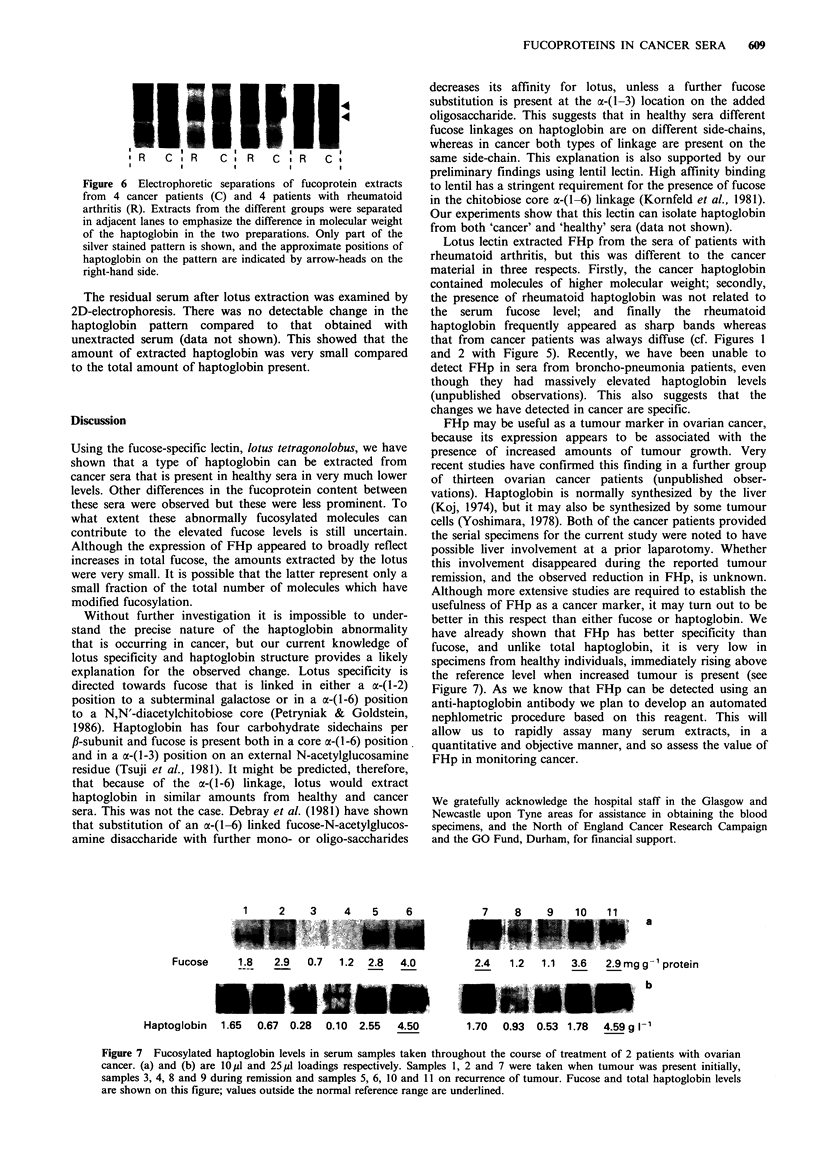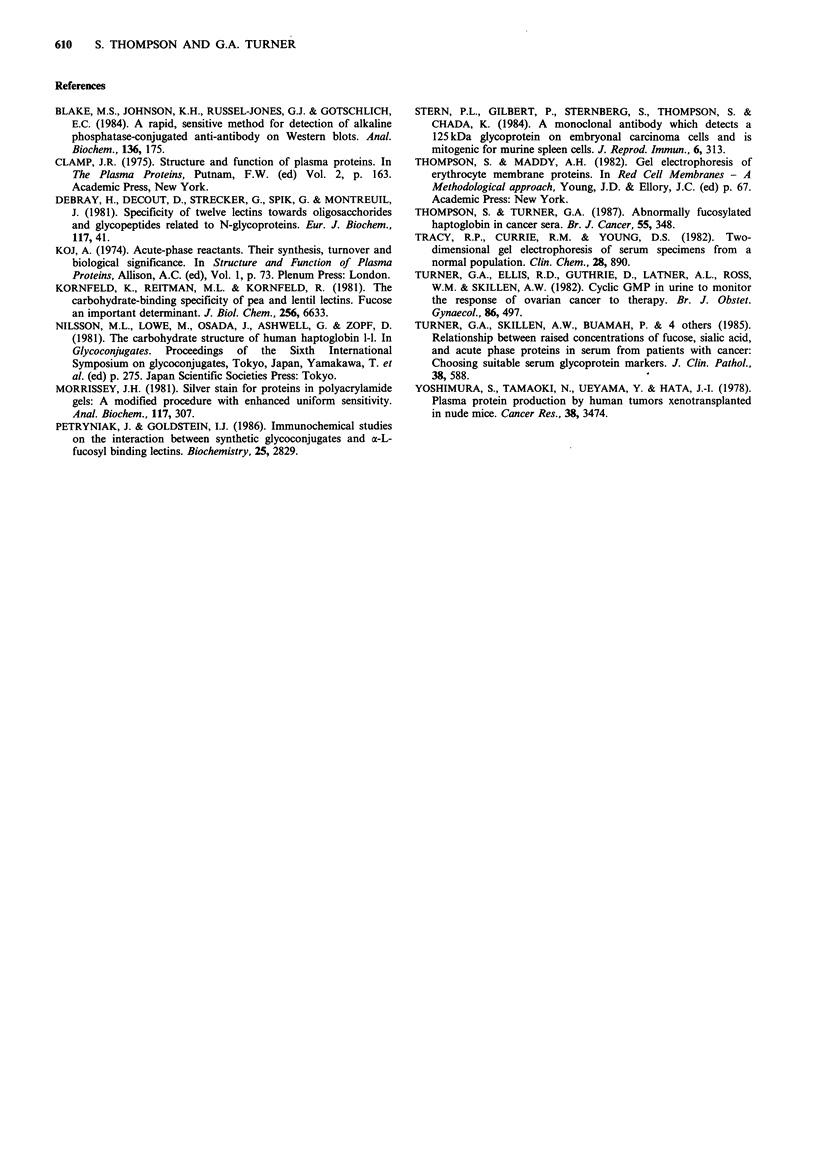# Elevated levels of abnormally-fucosylated haptoglobins in cancer sera.

**DOI:** 10.1038/bjc.1987.249

**Published:** 1987-11

**Authors:** S. Thompson, G. A. Turner

**Affiliations:** Department of Clinical Biochemistry, Medical School, Newcastle upon Tyne, UK.

## Abstract

**Images:**


*Br. J. Cancer* (1987) **56**, 605–610 © The Macmillan Press Ltd., 1987

### Elevated levels of abnormally-fucosylated haptoglobins in cancer sera

S. Thompson & G.A. Turner

*Department of Clinical Biochemistry, The Medical School, Newcastle upon Tyne, NE2 4HH, UK.*

**Summary** Cancer sera have higher levels of serum protein-bound fucose than sera from healthy individuals. In an attempt to identify the cause of this increase, fucoproteins were extracted from the sera of cancer patients and healthy individuals using a fucose-specific lectin (*lotus tetragonolobus*) coupled to Sepharose, and were analysed by polyacrylamide gel electrophoresis and silver staining. Of the several consistent changes observed for the cancer sera, the most striking was a large increase in a component of 40–45 Kdaltons. The expression of this component in the cancer sera was related to the elevation in serum fucose levels. Two dimensional (2D) electrophoretic analysis of lectin extracts showed that this component had a similar isoelectric point to the $\beta$ chains of haptoglobin. Its identity as haptoglobin was confirmed using Western blotting and an anti-haptoglobin antibody. Fucosylated haptoglobins (FHp) were also isolated from some rheumatoid arthritis sera, but there was no correlation between serum fucose levels and the FHp expression. The FHp in cancer sera was of higher molecular weight than that found in rheumatoid sera. Serial specimens from two ovarian cancer patients undergoing chemotherapy had elevated FHp associated with increased amounts of tumour. To the best of our knowledge this is the first time a molecule of this type has been reported in cancer sera and because of its uniqueness it deserves further investigation as a potential cancer marker.

Serum protein-bound focose is frequently elevated in cancer patients (Turner *et al.*, 1985). This finding cannot be explained by the production of new glycoproteins, as very few new proteins are seen on one (1D) or two dimensional (2D) electrophoresis of cancer sera as compared to the patterns of healthy sera (Thompson & Turner, unpublished observations). Other possible explanations include the increased production of pre-existing serum glycoproteins and/or alterations in the sugar moieties of these molecules. The acute phase proteins have been shown to be elevated in cancer (Turner *et al.*, 1985), but at their known normal levels of glycosylation (Clamp, 1975) it is unlikely that they can contribute much to the observed changes in fucose levels. Whether the fucosylation of serum proteins is altered in cancer is unclear, the objective of this study was to examine this possibility. The fucoprotein composition of sera from healthy individuals, cancer patients and arthritics has been investigated by using a fucose-specific lectin (lotus) in combination with electrophoresis. Some of the results have already been presented as a preliminary report (Thompson & Turner, 1987).

### Materials and methods

A single blood specimen was obtained from 22 cancer patients (12 men and 10 women; aged 17–67 years), 19 healthy volunteers (6 men and 13 women; aged 18–64 years) and 11 patients with rheumatoid arthritis (3 men and 8 women; aged 34–75 years) by venepuncture; separated by low speed centrifugation for 10 min, and the sera were stored at −20°C until required for analysis. The cancer group consisted of carcinomas unless stated otherwise. These were from the following sites: ovary 3; breast 3; teratoma 3; colon 2; lung 2; lymphoma 2; hepatoma 1; prostate 1; stomach 1; sarcoma 1; bile duct 1; kidney 1; melanoma 1. The cancer patients were either hospitalised or attending an outpatient clinic, were receiving a variety of chemotherapeutic treatments, and in the majority of cases the tumour had spread extensively and the disease was progressive; sera from this group will be subsequently designated as 'cancer'. The latter sera were chosen for the level of protein-bound fucose in their sera rather than for any particular clinical attribute. Two of the women with ovarian cancer provided serial specimens throughout their chemotherapy (cyclophosphamide), and during this time of collection they showed evidence of tumour remission followed by recurrence of tumour growth. The terms remission and recurrence have been previously defined (Turner *et al.*, 1982). Healthy volunteers were all individuals who attended a blood transfusion session, they had no known disease present and none of them were on medication or oral contraceptives; this group will be subsequently designated as 'healthy'. All the rheumatoid patients were attending an outpatient clinic, 5 had active disease and 8 were receiving medication; this group will be subsequently designated as 'rheumatoid'.

Lotus-lectin (*lotus tetragonolobus*; Sigma Chemical Co. Ltd., Poole, Dorset, UK) was coupled to CNBr activated Sepharose 4B beads (Pharmacia Ltd., Milton Keynes, UK) at a final concentration of 2 mg lectin $ml^{-1}$ packed beads using the method described in the Pharmacia handbook *Affinity Chromatography*. Immediately prior to use, the lectin-beads were washed three times with 2.5 ml 0.05 $mol\,l^{-1}$ Tris-HCl buffer, pH 7.4 containing 25 $mmol\,l^{-1}$ KCl, 5 $mmol\,l^{-1}$ $CaCl_2$, 5 $mmol\,l^{-1}$ $MgCl_2$ and 0.5% (v/v) Nonidet P40. One volume of packed lectin-beads (50–100 $\mu$l) was mixed with one volume of serum (50–100 $\mu$l) in a 3 ml plastic tube (LP3-Luckham Ltd., Burgess Hill, Sussex, UK) for 30–40 min at 25°C or for 1–2 h at 4°C. The reaction tube was gently agitated by hand every 10 min to ensure mixing of beads and serum. The beads were then washed 6 times (natural settling or gentle centrifugation at 4°C) with the above Tris-buffer to remove unbound serum components.

In pilot studies, bound glycoproteins were removed from the lectin-beads by either incubation for 30 min with 50–100 $\mu$l of the Tris washing buffer containing 0.5 $mol\,l^{-1}$ fucose, or by solubilising in 50–100 $\mu$l of 125 $mmol\,l^{-1}$ Tris HCl buffer pH 6.8, containing 0.35 $mol\,l^{-1}$ sodium dodecyl sulphate (SDS), 2.7 $mol\,l^{-1}$ glycerol, 1 $mmol\,l^{-1}$ EDTA, 2.9 $mmol\,l^{-1}$ bromophenol blue. Both these elution methods gave similar protein staining patterns after electrophoresis, but SDS appeared to elute all the components in greater amounts, therefore, this method was used to prepare the material for 1D-electrophoretic analyses. In 2D-analyses, material was always eluted with fucose because SDS interferes with the electrofocusing step. Two lectins with fucose affinity (lotus and gorse) were originally screened for their reactivity with serum glycoproteins, but lotus lectin was eventually chosen for this study because the extracted glycoproteins gave a clearer and more reproducible pattern on electrophoresis.

Correspondence: G.A. Turner.
Received 13 February 1987; and in revised form, 17 July 1987.

Serum glycoproteins and fucoprotein extracts were analysed by 1D and 2D polyacrylamide gel electrophoresis as previously described (Thompson & Maddy, 1982). Prior to 1D-electrophoresis, SDS-eluted material, or fucose-eluted material mixed with an equal volume of the SDS buffer, was reduced by the addition of $\beta$-mercaptoethanol to a final concentration of 0.72 mol $l^{-1}$ and boiling for 5 min. All eluates (10–30 $\mu$l/SDS; 20–50 $\mu$l/focuse) were then separated in 8% (w/v) polyacrylamide gels. Electrophoresis was carried out for 3.5 h at 40 mA/slab using the discontinuous Laemmli buffer system. For 2D-electrophoresis, 0.5 $\mu$l of unfractionated serum or 20 $\mu$l of the fucose eluate was separated by isoelectro-focusing (500 V for 20 h) in pH 3.5–10 gradients in 4% (w/v) polyacrylamide tube gels. After equilibration in sample buffer containing 5% (w/v) SDS, the tube gels were electrophoresed in a 8% (w/v) polyacrylamide second dimension. Separated proteins were stained with silver using a procedure similar to that described by Morrissey (1981). The latter method was modified by prefixing the gels in a solution containing 4.4 mol $l^{-1}$ ethanol and 1.7 mol $l^{-1}$ acetic acid for 2 h at 25°C; leaving out the glutaraldehyde step and increasing the dithiothreitol and silver nitrate treatment steps to 2 h. These modifications reduced the background staining and gave more reproducible results. Molecular weight markers were RNA polymerase (165, 155 and 39 Kdaltons (Kd), phosphorylase B (94 Kd) and serum albumin (68 Kd).

Western blotting and detection of antigen-antibody alkaline phosphatase-conjugated anti-antibody complexes were carried out by the method of Blake *et al.* (1984). Rabbit anti-human haptoglobin (Dakopatts) was used at a dilution of 1/1000 as the first layer and alkaline phosphatase-sheep anti-rabbit (Serotec) was used at the same dilution as the second layer.

Serum fucose and haptoglobin levels were measured using previously described methods (Turner *et al.*, 1985).

### Results

Figure 1 compares the 1D-electrophoresis patterns of fucosylated serum glycoproteins isolated from three cancer patients with those isolated from three age and sex matched healthy volunteers. At the bottom of this figure and Figures 2, 4, 5 and 7 are given the levels of serum protein-bound fucose. The particular 'cancer' sera shown in Figure 1 were investigated because of their high levels of serum fucose. Several reproducible differences can be seen between the 'healthy' and 'cancer' samples, but the largest difference is the increased expression of a 40–45 Kd glycoprotein in the 'cancer' extracts.

Figure 2 shows the 1D-patterns of lectin extracts from 12 'cancer' sera. These were analysed in two runs of 6 specimens per run. Two of the specimens shown in Figure 2a (specimens with fucose levels '4.6' and '4.8') are the same as the ones shown in Figure 1. These are included to illustrate the reproducibility of the analytical method. It can be seen from the data in Figure 2 that the elevation in the 40/45 Kd fucoprotein is consistently detected in 'cancer' sera that have an elevated fucose level. For two of the specimens in Figure 2a, the 40/45 Kd band was hardly detected; however, in one case the fucose level was below the upper limit of the normal range (1.8 mg $g^{-1}$ serum protein: Turner *et al.*, 1985), and in the other, the level was just above the normal range.

A 2D-separation of a lectin extract from a typical 'cancer' sera is shown in Figure 3. The position of the 40/45 Kd molecules is indicated by a large arrow-head where there appears to be two sets of strongly staining spots that focus between pH 4.8 and 5.8. These components were tentatively identified as the $\beta$ subunits of haptoglobin because of their position on the 2D gel. Haptoglobin has 2 $\beta$ subunits (Mr $\sim$ 40 Kd) and 2 $\alpha$ subunits (Mr $\sim$ 10 Kd) (Nilsson *et al.*, 1981) and the $\beta$ subunits focus over a similar pH range (Tracy *et al.*, 1982) to that observed for the extracted 40/45 Kd molecules.

The identity of the 40–45 Kd molecule as $\beta$ subunits of haptoglobin was confirmed in two ways. Firstly, an eluate from a lectin extract of a 'cancer' sera with high fucose levels was radioiodinated as described previously (Stern *et al.*, 1984), mixed with cold proteins from a 'healthy' serum and separated by 2D-electrophoresis. The radioactive 40–45 Kd molecules from this mixture ran in the same position as the silver stained haptoglobin molecules ($\beta$ subunits) of the unextracted serum (data not shown).

Secondly, a Western blot of a 1D-separation of 4 'healthy' and 7 'cancer' extracts was subsequently treated with an anti-haptoglobin antibody (Figure 4). Below the blot is presented the 40/45 Kd portion from another 1D-separation in which the same samples were silver stained. The particular 'cancer' specimens analysed were chosen to cover a wide range of serum fucose concentrations, and the data are presented for ascending fucose levels. It can be seen that the antibody detects a strong diffuse band at 40–45 Kd in the 'cancer' sera with highly elevated fucose levels ('3.3' and '3.2'). Even 'cancer' sera with less elevated fucose levels ('1.9', '2.0', and '2.4') show a shift in the position of the haptoglobin band to higher molecular weights and/or an increase in intensity compared with the 'healthy' sera.

Figure 5 compares the anti-haptoglobin blots of fucoproteins isolated from 6 'healthy' and 5 'rheumatoid' sera; a silver-stained inset is also shown below. A further 6 'rheumatoid' sera extracts were analysed by silver staining (data not shown). Elevated levels of fucosylated haptoglobins (FHp) were detected in 6 out of 11 of the rheumatoid specimens. The molecular weights of these species were lower

**Figure 1** Electrophoretic separation of fucoprotein extracts of sera (25 $\mu$l of extract loaded) from 3 healthy volunteers and 3 cancer patients. Changes in the composition of extracts of the 'cancer' group are indicated by solid triangles (increases) and open triangles (decreases). In **Figures 1, 2, 4, 5** and **7**; serum protein-bound fucose levels are given below the separations in mg fucose $g^{-1}$ protein; the positions of the mol. wt markers are indicated at the side of the patterns. In **Figures 1, 4, 5**; N or Normal = 'healthy'. The largest difference between the healthy and the cancer groups (40–45 Kd) is shown by a large arrow on the right hand side of the gel; this band is similarly indicated on **Figure 2**.

**Figure 2** Electrophoretic separations of fucoprotein extracts from 12 'cancer' patients. Aliquots of 30 $\mu$l (a) and 15 $\mu$l (b) were separated in two different experiments with two different batches of lectin. Background contaminants between 50–70 Kd are stained more heavily in (b) because the stain was developed longer due to lower sample loading.

**Figure 3** 2D-electrophoretic pattern of a lectin extract from a 'cancer' serum. The position of the 40–45 Kd molecules is indicated by a large arrowhead. First dimension (1) = electrofocusing; Second dimension (2) = electrophoresis.

than those observed for the 'cancer' sera (cf. Figures 4 and 5). This was further confirmed by the data in Figure 6 which shows part of a 1D-separation of 4 pairs of 'cancer' and 'rheumatoid' extracts that were separated in adjacent lanes. The difference in the mobility of FHp in the pairs of specimens is clearly evident. There was no relationship between the serum fucose level and FHp expression in the 'rheumatoid' extracts, but there was an association between elevated FHp and those individuals that had active disease at the time of taking the blood specimen. FHp was not elevated in the 'healthy' extracts analysed in Figure 5.

Figure 7 shows the content of FHp in serial serum specimens from two women with ovarian cancer who were undergoing chemotherapy. The specimens were taken on 5 or 6 occasions; 1 or 2 at the start of chemotherapy when tumour burden was high; 2 during clinical remission when tumour burden was very low; and finally 2 when a recurrence of tumour growth had occurred. Only the FHp regions of the 1D-patterns are shown with serum fucose and haptoglobin levels also listed. Results are given for two different sample loadings. Values of fucose or haptoglobin outside the normal reference range are underlined. For both patients, the expression of FHp and the elevation of fucose was correlated with the presence of increased amounts of tumour. Levels of haptoglobin also correlated with the presence of more tumour, but only one measurement for each patient was above the normal reference range ($>3.3\,g\,l^{-1}$: Turner *et al.*, 1985).

Other experiments were carried out to determine the amount of fucosylated glycoproteins removed from sera by extraction with lotus lectin. Ten sera (5 'healthy' and 5 'cancer') were extracted with either lotus-coupled Sepharose beads or control inactive beads, as described in the experimental section. The 'cancer' sera that were used all had elevated fucose levels. After treatment, fucose levels were measured in the sera (extracted and controls). Lotus treatment removed on average, 0.17 mg fucose $g^{-1}$ protein (SD = 0.04) from the 'healthy' sera and 0.19 mg fucose $g^{-1}$ protein (SD = 0.03) from the 'cancer' sera; this represented 13.8% and 6.4% of the total fucose in the respective sera prior to extraction.

**Figure 4** An anti-haptoglobin Western blot comparing 4 'healthy' and 7 'cancer' fucoprotein extracts (30 $\mu$l of extract loaded). An inset is shown below the blot which is the portion of a silver stained gel corresponding to the haptoglobin region of the same samples.

**Figure 5** An anti-haptoglobin Western blot of 6 'healthy' and 5 'rheumatoid' fucoprotein extracts (20 $\mu$l of extract loaded), compared to the silver stained haptoglobin region of the same samples (inset).

**Figure 6** Electrophoretic separations of fucoprotein extracts from 4 cancer patients (C) and 4 patients with rheumatoid arthritis (R). Extracts from the different groups were separated in adjacent lanes to emphasize the difference in molecular weight of the haptoglobin in the two preparations. Only part of the silver stained pattern is shown, and the approximate positions of haptoglobin on the pattern are indicated by arrow-heads on the right-hand side.

The residual serum after lotus extraction was examined by 2D-electrophoresis. There was no detectable change in the haptoglobin pattern compared to that obtained with unextracted serum (data not shown). This showed that the amount of extracted haptoglobin was very small compared to the total amount of haptoglobin present.

### Discussion

Using the fucose-specific lectin, *lotus tetragonolobus*, we have shown that a type of haptoglobin can be extracted from cancer sera that is present in healthy sera in very much lower levels. Other differences in the fucoprotein content between these sera were observed but these were less prominent. To what extent these abnormally fucosylated molecules can contribute to the elevated fucose levels is still uncertain. Although the expression of FHp appeared to broadly reflect increases in total fucose, the amounts extracted by the lotus were very small. It is possible that the latter represent only a small fraction of the total number of molecules which have modified fucosylation.

Without further investigation it is impossible to understand the precise nature of the haptoglobin abnormality that is occurring in cancer, but our current knowledge of lotus specificity and haptoglobin structure provides a likely explanation for the observed change. Lotus specificity is directed towards fucose that is linked in either a α-(1-2) position to a subterminal galactose or in a α-(1-6) position to a N,N′-diacetylchitobiose core (Petryniak & Goldstein, 1986). Haptoglobin has four carbohydrate sidechains per β-subunit and fucose is present both in a core α-(1-6) position and in a α-(1-3) position on an external N-acetylglucosamine residue (Tsuji *et al.*, 1981). It might be predicted, therefore, that because of the α-(1-6) linkage, lotus would extract haptoglobin in similar amounts from healthy and cancer sera. This was not the case. Debray *et al.* (1981) have shown that substitution of an α-(1–6) linked fucose-N-acetylglucosamine disaccharide with further mono- or oligo-saccharides decreases its affinity for lotus, unless a further fucose substitution is present at the α-(1–3) location on the added oligosaccharide. This suggests that in healthy sera different fucose linkages on haptoglobin are on different side-chains, whereas in cancer both types of linkage are present on the same side-chain. This explanation is also supported by our preliminary findings using lentil lectin. High affinity binding to lentil has a stringent requirement for the presence of fucose in the chitobiose core α-(1–6) linkage (Kornfeld *et al.*, 1981). Our experiments show that this lectin can isolate haptoglobin from both 'cancer' and 'healthy' sera (data not shown).

Lotus lectin extracted FHp from the sera of patients with rheumatoid arthritis, but this was different to the cancer material in three respects. Firstly, the cancer haptoglobin contained molecules of higher molecular weight; secondly, the presence of rheumatoid haptoglobin was not related to the serum fucose level; and finally the rheumatoid haptoglobin frequently appeared as sharp bands whereas that from cancer patients was always diffuse (cf. Figures 1 and 2 with Figure 5). Recently, we have been unable to detect FHp in sera from broncho-pneumonia patients, even though they had massively elevated haptoglobin levels (unpublished observations). This also suggests that the changes we have detected in cancer are specific.

FHp may be useful as a tumour marker in ovarian cancer, because its expression appears to be associated with the presence of increased amounts of tumour growth. Very recent studies have confirmed this finding in a further group of thirteen ovarian cancer patients (unpublished observations). Haptoglobin is normally synthesized by the liver (Koj, 1974), but it may also be synthesized by some tumour cells (Yoshimara, 1978). Both of the cancer patients provided the serial specimens for the current study were noted to have possible liver involvement at a prior laparotomy. Whether this involvement disappeared during the reported tumour remission, and the observed reduction in FHp, is unknown. Although more extensive studies are required to establish the usefulness of FHp as a cancer marker, it may turn out to be better in this respect than either fucose or haptoglobin. We have already shown that FHp has better specificity than fucose, and unlike total haptoglobin, it is very low in specimens from healthy individuals, immediately rising above the reference level when increased tumour is present (see Figure 7). As we know that FHp can be detected using an anti-haptoglobin antibody we plan to develop an automated nephlometric procedure based on this reagent. This will allow us to rapidly assay many serum extracts, in a quantitative and objective manner, and so assess the value of FHp in monitoring cancer.

We gratefully acknowledge the hospital staff in the Glasgow and Newcastle upon Tyne areas for assistance in obtaining the blood specimens, and the North of England Cancer Research Campaign and the GO Fund, Durham, for financial support.

**Figure 7** Fucosylated haptoglobin levels in serum samples taken throughout the course of treatment of 2 patients with ovarian cancer. (a) and (b) are 10 μl and 25 μl loadings respectively. Samples 1, 2 and 7 were taken when tumour was present initially, samples 3, 4, 8 and 9 during remission and samples 5, 6, 10 and 11 on recurrence of tumour. Fucose and total haptoglobin levels are shown on this figure; values outside the normal reference range are underlined.

### References

BLAKE, M.S., JOHNSON, K.H., RUSSEL-JONES, G.J. & GOTSCHLICH, E.C. (1984). A rapid, sensitive method for detection of alkaline phosphatase-conjugated anti-antibody on Western blots. *Anal. Biochem.*, **136**, 175.

CLAMP, J.R. (1975). Structure and function of plasma proteins. In *The Plasma Proteins,* Putnam, F.W. (ed) Vol. 2, p. 163. Academic Press, New York.

DEBRAY, H., DECOUT, D., STRECKER, G., SPIK, G. & MONTREUIL, J. (1981). Specificity of twelve lectins towards oligosacchorides and glycopeptides related to N-glycoproteins. *Eur. J. Biochem.*, **117**, 41.

KOJ, A. (1974). Acute-phase reactants. Their synthesis, turnover and biological significance. In *Structure and Function of Plasma Proteins,* Allison, A.C. (ed), Vol. 1, p. 73. Plenum Press: London.

KORNFELD, K., REITMAN, M.L. & KORNFELD, R. (1981). The carbohydrate-binding specificity of pea and lentil lectins. Fucose an important determinant. *J. Biol. Chem.*, **256**, 6633.

NILSSON, M.L., LOWE, M., OSADA, J., ASHWELL, G. & ZOPF, D. (1981). The carbohydrate structure of human haptoglobin 1-1. In *Glycoconjugates.* Proceedings of the Sixth International Symposium on glycoconjugates, Tokyo, Japan, Yamakawa, T. *et al.* (ed) p. 275. Japan Scientific Societies Press: Tokyo.

MORRISSEY, J.H. (1981). Silver stain for proteins in polyacrylamide gels: A modified procedure with enhanced uniform sensitivity. *Anal. Biochem.*, **117**, 307.

PETRYNIAK, J. & GOLDSTEIN, I.J. (1986). Immunochemical studies on the interaction between synthetic glycoconjugates and α-L-fucosyl binding lectins. *Biochemistry,* **25**, 2829.

STERN, P.L., GILBERT, P., STERNBERG, S., THOMPSON, S. & CHADA, K. (1984). A monoclonal antibody which detects a 125 kDa glycoprotein on embryonal carcinoma cells and is mitogenic for murine spleen cells. *J. Reprod. Immun.*, **6**, 313.

THOMPSON, S. & MADDY, A.H. (1982). Gel electrophoresis of erythrocyte membrane proteins. In *Red Cell Membranes – A Methodological approach,* Young, J.D. & Ellory, J.C. (ed) p. 67. Academic Press: New York.

THOMPSON, S. & TURNER, G.A. (1987). Abnormally fucosylated haptoglobin in cancer sera. *Br. J. Cancer,* **55**, 348.

TRACY, R.P., CURRIE, R.M. & YOUNG, D.S. (1982). Two-dimensional gel electrophoresis of serum specimens from a normal population. *Clin. Chem.*, **28**, 890.

TURNER, G.A., ELLIS, R.D., GUTHRIE, D., LATNER, A.L., ROSS, W.M. & SKILLEN, A.W. (1982). Cyclic GMP in urine to monitor the response of ovarian cancer to therapy. *Br. J. Obstet. Gynaecol.*, **86**, 497.

TURNER, G.A., SKILLEN, A.W., BUAMAH, P. & 4 others (1985). Relationship between raised concentrations of fucose, sialic acid, and acute phase proteins in serum from patients with cancer: Choosing suitable serum glycoprotein markers. *J. Clin. Pathol.*, **38**, 588.

YOSHIMURA, S., TAMAOKI, N., UEYAMA, Y. & HATA, J.-I. (1978). Plasma protein production by human tumors xenotransplanted in nude mice. *Cancer Res.*, **38**, 3474.